# P16^INK4A^ expression might be associated with a favorable prognosis for cervical adenocarcinoma via dysregulation of the RB pathway

**DOI:** 10.1038/s41598-021-97703-8

**Published:** 2021-09-14

**Authors:** Masako Ishikawa, Kentaro Nakayama, Kohei Nakamura, Hitomi Yamashita, Tomoka Ishibashi, Toshiko Minamoto, Kiyoka Sawada, Yuki Yoshimura, Kouji Iida, Sultana Razia, Noriyoshi Ishikawa, Satoru Nakayama, Yoshiro Otsuki, Satoru Kyo

**Affiliations:** 1 Department of Obstetrics and Gynecology, Shimane University Faculty of Medicine, Enyacho 89-1, Izumo, Shimane 6938501 Japan; 2 Department of Organ Pathology, Shimane University Faculty of Medicine, Izumo, 6938501 Japan; 3 Department of Obstetrics and Gynecology, Seirei Hamamatsu Hospital, Hamamatsu, 4308558 Japan; 4 Department of Organ Pathology, Seirei Hamamatsu Hospital, Hamamatsu, 4308558 Japan

**Keywords:** Cervical cancer, Cancer, Cancer genomics

## Abstract

Previous studies have largely failed to clarify the relationship between p16^INK4A^ status and cervical adenocarcinoma prognosis. The current study aimed to examine the clinical and pathological significance of p16^INK4A^ expression in several cervical adenocarcinoma subtypes. Eighty-two samples collected from patients with cervical adenocarcinoma were formalin fixed and paraffin embedded. Next, p16^INK4A^ levels were analyzed with immunohistochemistry. Additionally, the relationship between p16^INK4A^ expression and clinicopathological factors as well as prognosis was evaluated. The expression of p16^INK4A^ was mostly detected in all usual cervical adenocarcinoma subtypes. In the gastric type, only a few cases were positive for p16^INK4A^ expression. Results of the Kaplan–Meier analysis indicated that the positive p16^INK4A^ expression in tumor cells was significantly associated with favorable progression-free survival and overall survival in patients with cervical adenocarcinoma (*p* = 0.018 and *p* = 0.047, respectively, log-rank test). Our findings suggest that the status of p16^INK4A^ expression may influence prognosis. Thus, p16^INK4A^ expression could be used as a biomarker for improving the prognosis of patients with cervical adenocarcinoma.

## Introduction

According to a report of the National Cancer Institute, cervical cancer is the second most common cancer and the second leading cause of cancer-related deaths in women in their twenties and thirties^1^. Adenocarcinoma accounts for approximately 10‒25% of uterine cervical carcinoma cases^2–4^. Adenocarcinoma of the cervix is less common than squamous cell carcinoma (SCC), but the relative incidence of adenocarcinoma is increasing, particularly in young women^5–7^. Despite preventive measures, such as HPV vaccines and screening for cervical cancer via cytological examinations, adenocarcinoma is currently estimated to account for up to 25% of all cervical cancers^8,9^.

Currently, both these preventive measures are not effective for patients in Japan. Although a program encouraging vaccination was launched in June 2013, the HPV vaccine has since been withdrawn owing to many reports of adverse effects, such as complex regional pain syndrome (CPRS). Many junior high school students and their parents are wary of this syndrome, which has resulted in a decrease in new vaccination rates (0.97%) compared with those before these reports^10,11^. The World Health Organization (WHO) criticized the policy of the Japanese Government and declared that the government must resume vaccination for HPV; however, it has not been resumed. In Japan, the government recommends cervical cytology for cancer screening every 2 years for women from the age of 20 years. Nevertheless, monitoring of cervical cancer has been disregarded, leading to a cancer-screening rate of only 28.3% in 2016^12^. Thus, the prevention of cervical cancer in Japan has become problematic, and the development of new treatments and biomarkers for cervical cancer in a clinical setting is of utmost importance.

P16^INK4A^ acts as not only a surrogate marker of HPV infection but also a tumor suppressor gene, which regulates the cell cycle by specifically inhibiting cyclin D/CDK4/6 activity. Results of previous studies indicating a relationship between p16^INK4A^ expression and prognosis of cervical adenocarcinoma are considered controversial. Therefore, in the current study, we investigated the relationship between p16^INK4A^ expression and patient prognosis. Furthermore, we evaluated the potential relationship between p16^INK4A^ expression and immune-checkpoint inhibitor-related therapy in cervical adenocarcinoma.

## Materials and methods

### Tissue samples

Tissue samples were obtained from the Department of Obstetrics and Gynecology, Shimane University School of Medicine (Shimane, Japan) and Seirei Hamamatsu General Hospital (Shizuoka, Japan) between 2003 and 2017. Samples of all patients with cervical adenocarcinoma treated during the study period were included. The clinical information of patients was retrospectively obtained from electronic medical records.

The acquisition of tumor tissues was approved by the Institutional Review Board, Shimane University (IRB Nos. 20070305-1 and 20070305-2). After appropriate explanation, written informed consent was obtained from the patients for the procedure and for participation in the study. For those who could not visit the hospitals again, we had clearly announced that the opportunity to opt out of the study is always available to patients by taking measures such as providing information regarding opting out, on the website.

All experiments were performed in accordance with relevant guidelines and regulations. Furthermore, the study was performed in accordance with tenets of the Helsinki Declaration.

A total of 82 samples were collected from patients with uterine cervical adenocarcinoma who underwent surgical resection or biopsy. Diagnoses were confirmed by a gynecopathologist (IN) trained at our institution. Many patients were primarily treated with surgery, and most of them had received adjuvant therapies such as chemotherapy, radiotherapy, and concurrent chemoradiotherapy (CCRT) with a platinum drug (weekly cisplatin; 40 mg/m^2^). These treatment strategies were selected based on the Japanese guidelines for cervical cancer.

### Immunohistochemistry

The expression of p16^INK4A^ and immune escape mechanism-related factors (CD8, PD-L1, and PD-1) was evaluated with immunohistochemistry (IHC). Formalin-fixed and paraffin-embedded sections (4 μm thick) were dewaxed in xylene and hydrated via an alcohol gradient. Following antigen retrieval in a sodium citrate buffer, the sections were incubated overnight at 4 °C with antibodies against p16^INK4A^ (1:30; cloneE6H4; mtm-Roche Diagnostics, Heildelberg, Germany), CD-8 (1:100; Roche, Basel, Switzerland), PD-L1 (1:400; ab205921; Abcam, Cambridge, United Kingdom), and PD-1 (1:100; Roche). Samples were evaluated under a light microscope by a pathologist, who was blinded to clinicopathological factors.

### Definition of p16^INK4A^ and CD8/PD-1/ PD-L1 positivity by IHC

The expression of p16^INK4A^ in both cytoplasm and nucleus was evaluated by staining for three tumor density categories as follows: 0 (undetectable), 1 + (low density), and 2 + (high density). Intensities of + 1 and + 2 were considered as strong staining. In a previous study, over 70% of cervical cancer cells with strong p16 staining of the nuclei and cytoplasm were regarded as p16 positive^13^. Therefore, in the current study, most of the tumor cells with strong p16 staining were considered as p16 positive. The population density of tumor infiltrating lymphocytes was stratified by CD8 staining into three categories as follows: 0 (undetectable), 1 + (low density, 0–30%), and 2 + (moderate-high density, ≥ 30%). Samples that were categorized as 2 + were considered positive. For PD-L1, tumors with ≥ 5% of stained tumor cells (membranous and cytoplasmic staining) were considered positive. For PD-1, tumors with ≥ 5% of tumor-infiltrating stained lymphocytes were considered positive.

### Statistical analysis

The correlation between p16^INK4A^ expression and clinicopathological characteristics and patient prognosis was analyzed by chi-square test. Furthermore, the correlation between p16^INK4A^ expression and other immune escape mechanism-related factors was analyzed using the chi-squared test. Progression-free survival (PFS) and overall survival (OS) were analyzed using the Kaplan–Meier method with the log-rank test. The univariate and multivariate Cox proportional hazard regression analyses were followed by binomial logistic regression for ordered categorical variables. Statistical analyses were performed using IBM SPSS (IBM, Armonk, NY, USA), version 23. Statistical significance was set at *p* < 0.05.

### Ethics declaration

The acquisition of tumor tissues was approved by the Shimane University Institutional Review Board (IRB Nos. 20070305-1 and 20070305-2).

### Consent to participate

After appropriate explanation, patients provided written informed consent for the procedure and for participation in the study. For patients who could not visit hospitals again, we had clearly announced that the opportunity to opt out is always available by taking measures such as carrying the information on the opportunity to opt out on the website, as well as making arrangements so that patients can opt out via the website at any time.

## Results

### Clinicopathological characteristics of the patients

**Figure 1:**
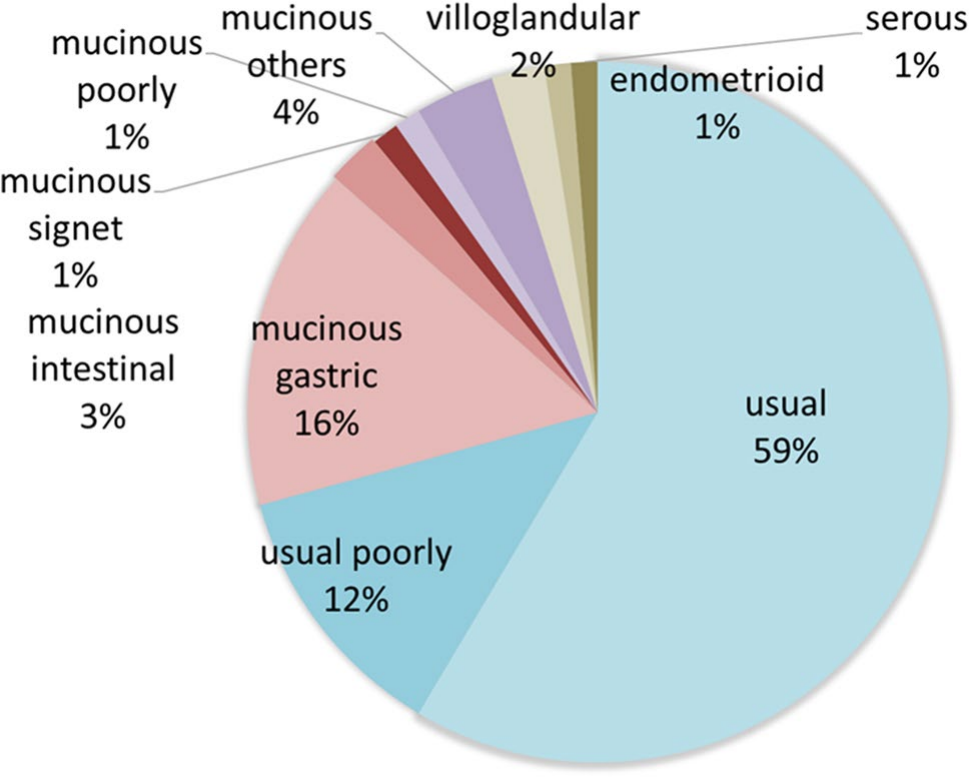
Rate of pathological subtypes with strong p16^INK4A^ expression.

Histological subtypes of the test cases are shown in Table 1 and Fig. 1.

**Table 1:** Relationship between p16^INK4A^ status and pathological subtype.

Subtype	Patients, n = 82	p16^INK4A^	
		Positive n = 60 (73.2%)	Negative n = 22 (26.8%)
Usual	48	40 (83.3)	8 (16.7)
Usual poorly	10	10 (100.0)	0
Mucinous gastric	13	2 (15.4)	11 (84.6)
Mucinous intestinal	2	1 (50.0)	1 (50.0)
Mucinous signet	1	1 (100.0)	0
Mucinous poorly	1	1 (100.0)	0
Mucinous others	3	2 (66.7)	1 (33.3)
Villoglandular	2	2 100.0)	0
Endometrioid	1	0	1 (100.0)
Serous	1	1 (100.0)	0

There were several histological subtypes in cervical adenocarcinoma, including the usual type, gastric type, and others. The usual type was the most popular type (59.0%), followed by the gastric type (16%). The clinicopathological characteristics of the test patients are summarized in Table 2 and Supplementary Table S1.

**Table 2:** Characteristics of patients with cervical adenocarcinoma.

Number of patients	n = 82
Age (years), median (range)	50.4 (30–85)
**FIGO stage n, (%)**	
IA	5 (6.1)
IB	48 (58.5)
IIA	5 (6.1)
IIB	14 (17.1)
IIIA	0
IIIB	8 (9.8)
IVA	0
IVB	2 (2.4)
**Tumor size (cm) median (range)**	32.7 (0–80)
Unknown	5
**Tumor size**	
< 4 cm	49 (59.6)
≥ 4 cm	33 (40.4)
**LVSI**	
Yes	39 (47.6)
No	29 (35.4)
Unknown	14 (17.0)
**Metastases pelvic LN**	
Yes	17 (20.7)
No	65 (79.3)
**Metastases paraaortic LN**	
Yes	3 (3.7)
No	79 (92.3)
**Metastases distance**	
Yes	1(1.2)
No	81 (98.8)
**Treatment**	
Surgery	38 (46.3)
Surgery + adjuvant (RT or CCRT or CT)	40 (48.8)
Radiotherapy (RT or CCRT)	4 (4.8)
Chemotherapy	0
**Recurrence within 5 years**	
Yes	20 (24.4)
No	60 (75.6)
**Death within 5 years**	
Yes	19 (23.2)
No	63 (76.8)

In the present study, we identified clinical stages according to the definition of the International Federation of Gynecology and Obstetrics (FIGO) 2014 as stages IA, IB1, B2, IIA, IIB, IIIB, and IVB. Treatments received by the test patients were as follows: 72 underwent radical hysterectomy and received adjuvant therapy, such as concurrent chemoradiotherapy (CCRT); 9 patients with advanced stage cancer received CCRT; and 1 patient received chemotherapy without surgery due to multiple distant metastases. Radiotherapy (whole pelvic irradiation) or chemotherapy (paclitaxel 175 mg/m^2^ and carboplatin area under the curve = 5 mg/m^2^) was administered postoperatively in patients with a high recurrence risk because of locally advanced stage, non-SCC type of histology, bulky tumor > 4 cm, deep infiltration depth of cervical tumor, grade 2 or 3, lymph node metastasis, or lympho-vascular space invasion.

The chemotherapy regimens adopted were paclitaxel plus carboplatin, paclitaxel plus cisplatin, docetaxel plus carboplatin, irrinotecan plus cisplatin, and gemcitabine. In the CCRT regimen, 5‒6 courses of 40 mg/m^2^ cisplatin were administered weekly.

### Relationship between p16^INK4A^ expression and clinicopathological factors in cervical adenocarcinoma

**Figure 2:**
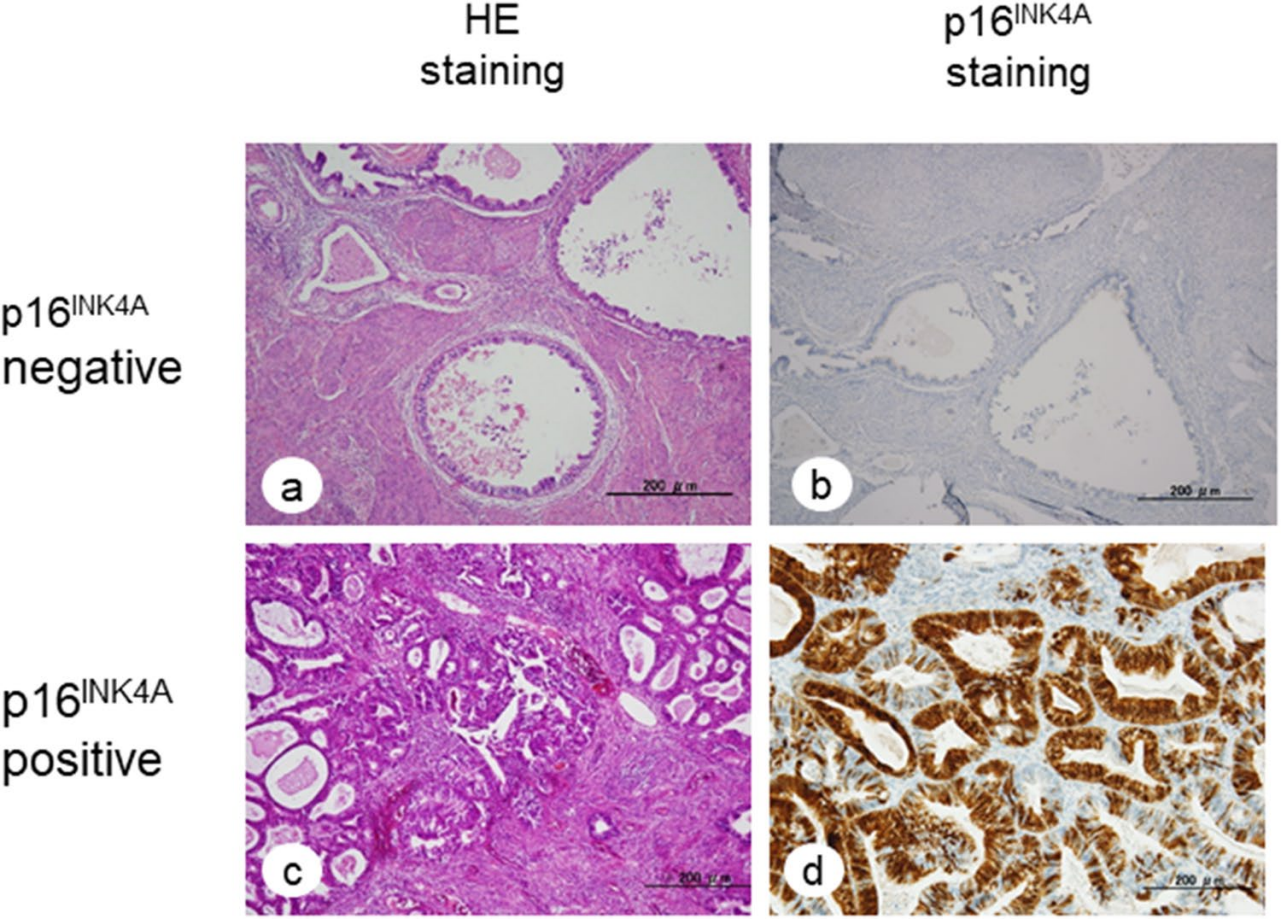
HE staining and immunohistochemistry of specimens obtained from patients with cervical adenocarcinoma. The expression of p16^INK4A^ was evaluated in three categories of tumor density via staining: 0 (undetectable); 1 + (low density); 2 + (high density). Cases that were 2 + were considered positive and those with 0 and + 1 were considered negative.

In the present study, 60/82 (73.1%) patients displayed strong p16^INK4A^ expression. Representative cases with positive or negative p16^INK4A^ expression are shown in Fig. 2. Significant relationships were observed between p16^INK4A^ expression and age (*p* = 0.001), FIGO stage (*p* = 0.002), histological subtype (*p* < 0.0001), pelvic lymph node metastasis (*p* = 0.034), LVSI metastasis (*p* = 0.003), and disease recurrence (*p* = 0.021) (Table 3).

**Table 3:** Relationship between p16^INK4A^ expression and the clinicopathological factors of patients with cervical adenocarcinoma.

Factors	Patients	p16^INK4A^		*p *value
	n = 82	Negative n, (%)	Positive n, (%)	
**Age (years)**				
< 60	58	10 (17.2)	48 (82.8)	0.002
> 60	24	12 (50.0)	12 (50.0)	
**FIGO stage**				
< IIB	58	10 (17.2)	48 (82.8)	0.002
≥ IIB	24	12 (50.0)	12 (50.0)	
**Histology**				
Non-gastric	69	11 (15.9)	58 (84.1)	< 0.0001
Gastric	13	11 (84.6)	2 (15.4)	
**Histology**				
Non-usual	22	14 (63.6)	8 (36.4)	< 0.0001
Usual	60	8 (13.3)	52 (86.7)	
**Metastasis Pelvic lymph node**				
Negative	65	14 (12.5)	51 (78.5)	0.034
Positive	17	8 (47.1)	9 (52.9)	
**Metastasis Paraaortic lymph node**				
Negative	79	21 (26.6)	58 (73.4)	0.796
Positive	3	1 (33.3)	2 (66.7)	
**LVSI ly**				
No	29	2 (6.9)	27 (93.1)	0.003
Yes	39	15 (38.5)	24 (61.5)	
**LVSI v**				
No	31	2 (6.5)	29 (93.5)	0.001
Yes	39	16 (41.0)	23 (59.0)	
**Tumor size (mm)**				
< 40	50	10 (20.4)	40 (79.6)	0.081
≥ 40	32	12 (37.5)	10 (62.5)	
**Recurrence**				
No	60	12 (20.0)	48 (80.0)	0.021
Yes	22	10 (45.5)	12 (54.5)	
**Died due to the disease**				
No	63	14 (22.2)	49 (77.8)	0.086
Yes	19	8 (42.1)	11 (57.9)	

### Relationships between p16^INK4A^ expression and CD8, PD-L1, and PD-1 expression in cervical adenocarcinoma

The relationships between p16^INK4A^ expression and CD8, PD-L1, or PD-1 expression were assessed using the chi-squared test. The positive rates of expression of immune checkpoint-related factors, such as CD8, PD-1, and PD-L1, were not significantly different according to p16^INK4A^ expression (Supplementary Table S2).

### Clinical features of cervical adenocarcinoma with p16INK4A expression

**Figure 3:**
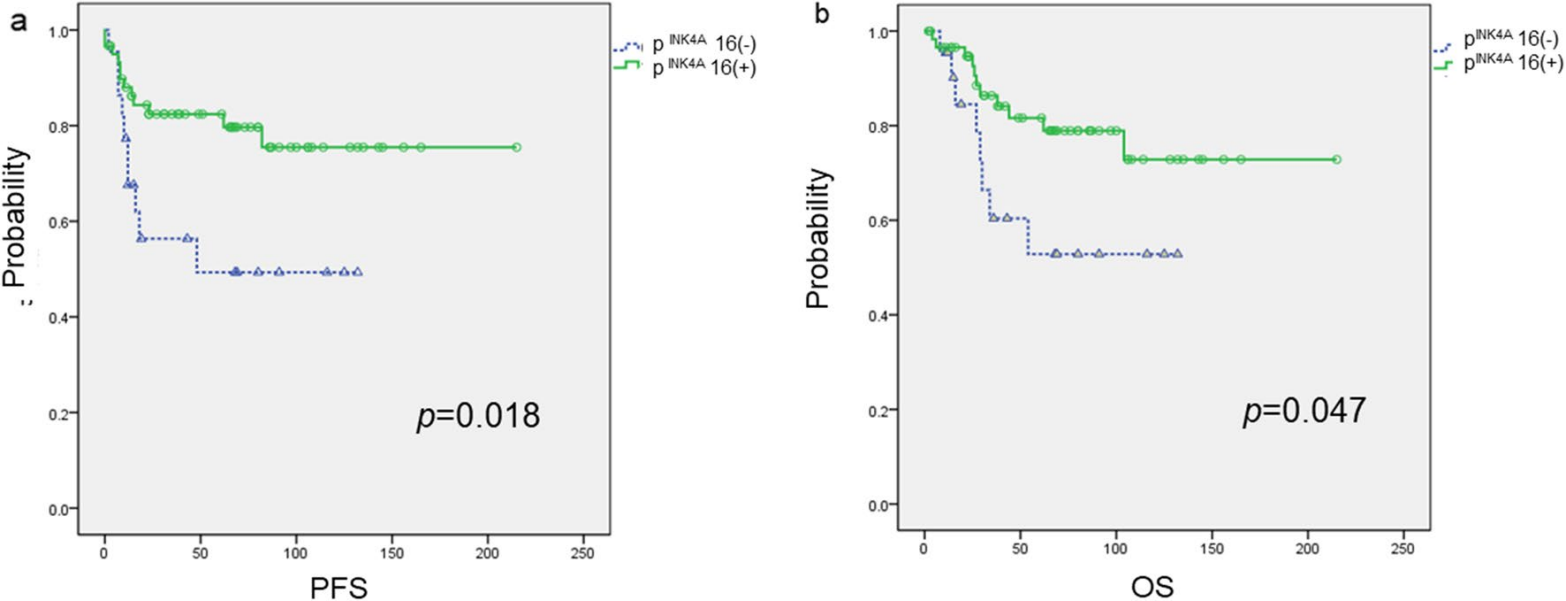
Kaplan–Meier analysis of progression-free (**a**) and overall (**b**) survival between the p16^INK4A^-positive and negative groups. PFS was significantly extended in the p16^INK4A^-positive group compared with that in the negative group (*p* = 0.018, log-rank test; **a**). OS was also extended in the p16^INK4A^-positive group compared with that in the negative group (*p* = 0.047, log-rank test; **b**).

Kaplan–Meier analysis was performed to determine the potential correlation between p16^INK4A^ expression and prognosis. Cervical adenocarcinoma patients with p16^INK4A^ negativity presented significantly worse PFS and OS than those with p16^INK4A^ positivity (*p* = 0.018 and *p* = 0.047, respectively, log-rank test; Fig. 3a,b).

### Univariate analysis of prognostic factors in patients with cervical adenocarcinoma

The univariate and multivariate Cox regression analyses of the prognostic factors in patients with cervical adenocarcinoma are shown in Tables 4 and 5. The univariate and multivariate logistic regression models were used for proportional hazards analysis of prognostic factors with a hazard ratio (HR) and 95% confidence interval.

**Table 4:** Univariate and multivariate analyses of progression-free survival using a Cox proportional hazards model in patients with cervical adenocarcinoma.

Factor	Patients	Univariate analysis			Multivariate analysis		
	n = 82	HR	95% CI	*p* value	HR	95% CI	*p* value
**Age (years)**							
< 60	58	0.552	0.236–1.293	0.171			
≥ 60	24	ref					
**FIGO stage**							
< IIB	58	0.194	0.081–0.464	< 0.0001	0.85	0.252–2.867	0.793
≥ IIB	24	ref			ref		
**Histology**							
Non-gastric type	69	0.819	0.277–2.422	0.819			
Gastric type	13	ref					
**Tumor size (mm)**							
< 40	50	0.135	0.050–0.369	0.0001	0.161	0.036–0.719	0.017
≥ 40	32	ref			ref		
**Metastasis Pelvic Lymph node**							
Negative	65	0.379	0.162–0.889	0.026	0.553	0.165–1.858	0.338
Positive	17	ref			ref		
**Metastasis Paraaortic Lymph node**							
Negative	79	0.209	0.061–0.707	0.012	ref		
Positive	3	ref			1.828	0.292–11.466	0.519
**Metastasis distance**							
Negative	81	0.078	0.009–0.644	0.018	0.503	0.047–5.320	0.568
Positive	1	ref			ref		
**Metastasis LSVI**							
No	29	0.453	0.087–2.353	0.004	0.065	0.007–0.597	0.016
Yes	39	ref			ref		
**p16**^**INK4A**^** expression**							
Negative	22	ref			ref		
Positive	60	0.376	0.162–0.873	0.023	0.965	0.331–2.812	0.948

**Table 5:** Univariate and multivariate analyses of overall survival using a Cox proportional hazards model in patients with cervical adenocarcinoma.

Factor	Patients	Univariate analysis			Multivariate analysis		
	n = 82	HR	95% CI	*p* value	HR	95% CI	*p* value
**Age (years)**							
< 60	58	0.58	0.233–1.446	0.243			
≥ 60	24	ref					
**FIGO stage**							
< IIB	58	0.222	0.087–0.565	0.002	0.912	0.253–3.289	0.889
≥ IIB	24	ref			ref		
**Histology**							
Non-gastric type	69	0.67	0.222–2.021	0.477			
Gastric type	13	ref					
**Tumor size (mm)**							
< 40	50	0.168	0.060–0.468	0.001	0.251	0.059–1.078	0.063
≥ 40	32	ref			ref		
**Metastasis Pelvic Lymph node**							
Negative	65	0.515	0.202–1.309	0.163			
Positive	17	ref					
**Metastasis Paraaortic Lymph node**							
Negative	79	0.321	0.074–1.405	0.131			
Positive	3	ref					
**Metastasis distance**							
Negative	81	0.026	0.002–0.283	0.003	0.095	0.008–1.170	0.066
Positive	1	ref			ref		
**Metastasis LVSI**							
No	29	0.053	0.007–0.416	0.005	0.06	0.006–0.571	0.014
Yes	39	ref			ref		
**p16**^**INK4A**^** expression**							
Negative	22	ref			ref		
Positive	60	0.409	0.164–1.020	0.055	0.951	0.295–3.064	0.933

The results of univariate analysis indicated a significant correlation between PFS and p16^INK4A^ expression (HR: 0.376, p5%; CI 0.162‒0.873, *p* = 0.023) (Table 4). A significant correlation was also observed between PFS and FIGO stage, LVSI, tumor size, and metastasis of pelvic lymph node, paraaortic lymph node, or distance. The multivariate analysis revealed a significant correlation between PFS and LVSI or tumor size. We also performed a stratified multivariate analysis in early-stage cases; however, no significant correlation was observed between PFS and p16 expression (Supplementary Table S3). The univariate analysis revealed a significant correlation between OS and FIGO stage, LVSI, or tumor size, and the multivariate analysis demonstrated a significant correlation between LVSI and OS.

## Discussion

This study demonstrated that strong p16^INK4A^ expression is related to a favorable prognosis of cervical adenocarcinoma. In contrast, the results of previous studies on the association between p16^INK4A^ expression and cervical adenocarcinoma have been unclear^13–15^.

The 2018 International Endocervical Adenocarcinoma Criteria and Classification (IECC) distinctively explained the criteria pertaining to the pathological diagnosis of cervical adenocarcinoma, compared with the WHO criteria^16^. According to the IECC, diagnosis was defined using the HPV infection status, and it reflected patient prognosis. Furthermore, they substantiated their opinion by publishing clinical outcomes of patients with cervical adenocarcinomas associated or unassociated with HPV infection^17^. In the current study, we replicated their results. At the start of this study, we assumed that tumors with strong p16^INK4A^ expression predicted good prognosis because p16^INK4A^ is a surrogate marker of HPV infection. As viruses express antigens, lymphocytes expressing CD8 attack cancer cells^18^. According to our predictions, cervical adenocarcinoma patients with strong p16^INK4A^ expression showed the trend of favorable prognosis. However, a positive relationship between p16^INK4A^ expression and the expression of immune-check point-related molecules, such as CD8, PD-1, and PD-L1, was not verified (Supplementary Table S2). Previously, we reported the association between cervical adenocarcinoma and immune characteristics^19^. We demonstrated that a high PD-1 expression may be associated with a poor prognosis in patients with cervical adenocarcinoma. However, in that study, we did not analyze the relationship between the expression of p16 and immune-check point-related molecules. The lack of a significant positive relationship between p16^INK4A^ expression and immune-check point-related molecules can be attributed to the fact that cervical adenocarcinomas with HPV infection possibly do not continue to express p16^INK4A^ following malignant alteration. A previous study reported that some patients with cervical adenocarcinoma have reduced expression of p16 when the tumor is malignant^20^. In the current study, the positive rate of p16^INK4A^ expression was considerably higher in early-stage cervical adenocarcinoma than in advanced-stage cervical adenocarcinoma (Table 3).

Several studies have reported that p16^INK4A^ overexpression may be considered as a surrogate marker for high-risk HPV infection in the cervix^21–23^. As HPV infection induces tumor immune-environmental activity, immunotherapy has been recognized as an attractive treatment strategy for cervical carcinoma, as for other malignant tumors^24^.

Although carcinogenesis is associated with HPV infection, these tumors do not maintain steady p16^INK4A^ (HPV infection) expression during carcinogenesis and tumor growth. The expression of p16^INK4A^ changes under varying conditions, and there are currently no markers associated with the loss of p16 expression in some HPV-positive tumors before confirming malignant tumors. In addition, microenvironmental immunoactivity around HPV-infected tumors has not yet been conclusively proven, owing to which an association between the condition (stage, tumor size, and propensity of invasion) of the tumors themselves and the function of immune-related lymphocytes has not been demonstrated. Thus, further examination may be required to assess the changes in microenvironmental immunoactivity around tumors that have been already infected with HPV and have outgrown their previous conditions.

Another possibility is that p16^INK4A^ also functions as a tumor suppressor gene, and the loss of p16^INK4A^ accentuates the phosphorylation of the retinoblastoma (RB) protein (pRB). This pathway may be more pathophysiologically important. P16^INK4A^ is a tumor suppressor gene, and it regulates the cell cycle by specifically inhibiting the cyclin D/CDK4/6 activity. P16^INK4A^ and pRB form a negative feedback loop and the inactivation or mutation of pRB leads to the overexpression of p16^INK4A^, resulting in CDK4 and CDK6 dysregulation^25,26^.

Therefore, cell proliferation is suppressed and tumors that express p16^INK4A^ would have a good prognosis. Llucia et al. further indicated that p16^INK4A^ expression reflected not only the status of HPV infection but also dysregulation of the RB pathway, particularly in head and neck malignant tumors^27^.

Overall, the findings of previous studies and the current study indicate that p16^INK4A^ expression may be a favorable prognostic factor, associated with the pRB pathway, rather than with HPV infection, in cervical adenocarcinoma. Several studies have indicated that immunocyte invasion by HPV infection may contribute to the effectiveness of its treatment, whereas p16^INK4A^ expression reflects tumor suppressor properties, as well as its role in inhibiting CDK4 and maintaining pRB. Missaoui et al. reported that p16^INK4A^ expression primarily affects the RB protein-related pathway, rather than the HPV-independent pathway^28^. In the future, we aim to study mechanism(s) underlying the changes in p16^INK4A^ expression at the onset of HPV infection, during tumorigenesis and tumor growth. We will also investigate the changes in the expression of immune-related factors associated with tumor condition and p16^INK4A^ expression.

Another notable point is that the prognosis of gastric type was very poor. Mucinous gastric type was associated with a very poor prognosis; however, we could not unravel the mechanism underlying the correlation between poor prognosis and negative HPV infection in gastric-type tumors. The expression of p16 could be affected by various factors. We want to emphasize that p16-negative tumor subtypes such as gastric-type tumors are not associated with HPV infection and show a poor prognosis because of the inactivation of a tumor suppressor gene. Currently, we are conducting genetic analysis of gastric-type tumor using whole-exome sequencing.

Our study had some limitations. Our series of cervical adenocarcinomas with confirmed p16^INK4A^-negativity were frequently found in an advanced FIGO stage, showing a higher rate of lymph node metastasis. Therefore, the possibility of better responses of p16^INK4A^-positive cases in advanced FIGO stage to chemotherapy and radiation therapy, as observed in head and neck cancer cases^27,29^, cannot be excluded. Availability of a higher number of cases may have allowed comparisons within the same FIGO stage, leading to more information regarding each p16^INK4A^ condition.

A further limitation was that only p16^INK4A^ expression was examined. Addition of PCR analyses or IHC of HPV may have aided in the clarification of the precise relationship between p16^INK4A^ expression and HPV infection status. However, some studies have reported that while p16^INK4A^ expression reflects HPV infection^30^, it may not necessarily reflect tumors with HPV infection^31^. Due to difficulties in correctly and easily establishing whether a tumor has HPV infection, only p16^INK4A^ expression was taken into consideration.

In summary, results of the current study indicated that p16^INK4A^ expression might be associated with favorable outcomes in patients with cervical adenocarcinoma. Thus, p16INK4A expression could serve as a biomarker for improving the prognosis of patients with cervical adenocarcinoma. Further research is needed to clarify that the expression of p16^INK4A^ may function as a tumor suppressor rather than an HPV infection suppressor, activating invasive immune system cells.

## Supplementary Information

Supplementary Information.

## Data Availability

Data of current study was available from corresponding author (K.N.).
