# Supplementary material for: P16INK4A expression might be associated with a favorable prognosis for cervical adenocarcinoma via dysregulation of the RB pathway
Source: Sci Rep. 2021 Sep 14;11:18236. doi: 10.1038/s41598-021-97703-8 (PMC8440605; doi:10.1038/s41598-021-97703-8)
Supplement: Supplementary file 1 — Supplementary Information. [file 41598_2021_97703_MOESM1_ESM.docx]

**P16^INK4A^ expression might be associated with a favorable prognosis for cervical adenocarcinoma via dysregulation of the RB pathway**

Masako Ishikawa, Kentaro Nakayama, Kohei Nakamura, Hitomi Yamashita, Tomoka Ishibashi, Toshiko Minamoto, Kiyoka Sawada, Yuki Yoshimura, Kouji Iida, Sultana Razia, Noriyoshi Ishikawa, Satoru Nakayama, Yoshiro Otsuki, and Satoru Kyo

Supplementary Table 1. Characteristics of patients with cervical adenocarcinoma (stratification by age)

| Stratification by age (years) | 30–85 | 30–59 | 60–74 | 75–85 |
| --- | --- | --- | --- | --- |
| Number of patients | n = 82 | n = 58 | n = 17 | n = 7 |
|  |  |  |  |  |
| Age (years), median (range) | 50.4 (30–85) | 42.8 (30–59) | 64.5 (60–74) | 78.9 (75–85) |
|  |  |  |  |  |
| FIGO stage n, (%) |  |  |  |  |
| ⅠA | 5 (6.1) | 5 (8.6) | 0 | 0 |
| ⅠB | 48 (58.5) | 40 (69.0) | 7 (41.2) | 1 (14.3) |
| ⅡA | 5 (6.1) | 4 (6.9) | 1 (5.9) | 0 |
| ⅡB | 14 (17.1) | 8 (13.8) | 7 (4.1) | 0 |
| ⅢA | 0 | 0 | 0 | 0 |
| ⅢB | 8 (9.8) | 1 (1.7) | 2 (11.8) | 4 (57.1) |
| ⅣA | 0 | 0 | 0 | 0 |
| ⅣB | 2 (2.4) | 0 | 0 | 2 (28.6) |
|  |  |  |  |  |
| Tumor size (cm) median (range) | 32.7 (0–80) | 28.3 (0–72) | 30.7 (0–80) | 46.5 (0–66) |
|  |  |  |  |  |
|  |  |  |  |  |
| Tumor size |  |  |  |  |
| <4 cm | 49 (59.6) | 40 (69.0) | 8 (47.1) | 2 (28.6) |
| >4 cm | 33 (40.4) | 18 (31.0) | 9 (52.9) | 5 (71.4) |
|  |  |  |  |  |
| LVSI |  |  |  |  |
| Yes | 39(47.6) | 22 (37.9) | 12 (70.6) | 5 (71.4) |
| No | 29 (35.4) | 26 (44.8) | 3 (17.6) | 0 |
| unknown | 14 (17.0) | 10 (17.2) | 2 (11.8) | 2 (28.6) |
|  |  |  |  |  |
| Metastases pelvic LN (On CT imaging) |  |  |  |  |
| Yes | 17 (20.7) | 9 (15.5) | 7 (41.2) | 1 (14.3) |
| No | 65 (79.3) | 49 (84.5) | 10 (58.8) | 6 (85.7) |
|  |  |  |  |  |
| Metastases paraaortic LN (On CT imaging) | |  |  |  |
| Yes | 3 (3.7) | 2 (3.4) | 1 (5.9) | 0 |
| No | 79 (92.3) | 56 (96.6) | 16 (94.1) | 7 |
|  |  |  |  |  |
| Metastases distance (On CT imaging) |  |  |  |  |
| Yes | 2(2.4) | 0 | 0 | 2 (28.6) |
| No | 81(97.6) | 58 | 17 | 5 (71.4) |
|  |  |  |  |  |
| Treatment |  |  |  |  |
| Surgery | 38 (46.3) | 22 (37.9) | 3 (17.6) | 2 (28.6) |
| Surgery+adjuvant (RT or CCRT or CT) | 40 (48.8) | 33 (56.9) | 9 (52.9) | 2 (28.6) |
| Radiotherapy (RT or CCRT) | 4 (4.8) | 2 (3.4) | 5 (29.4) | 2 (28.6) |
| Chemotherapy | 0 | 0 | 0 | 1 (14.3) |
|  |  |  |  |  |
| Recurrence within 5 years |  |  |  |  |
| Yes | 20 (24.4) | 13 (22.4) | 5 (29.4) | 4 (57.1) |
| No | 60 (75.6) | 45 (77.6) | 12 (70.6) | 3 (42.9) |
|  |  |  |  |  |
| Death within 5 years |  |  |  |  |
| Yes | 19 (23.2) | 11 (19.0) | 4 (23.5) | 4 (57.1) |
| No | 63 (76.8) | 47 (81.0) | 13 (76.5) | 3 (42.9) |

| Supplementary Table 2. Relationship between PD-1 expression and the clinicopathological factors of patients with cervical adenocarcinoma | | | | | | | |
| --- | --- | --- | --- | --- | --- | --- | --- |
|  |  |  |  |  |  |  |  |
| Parameter | p16 positive | p16 negative | *p*-value |  |  |  |  |
|  | n = 60 | n = 22 |  |  |  |  |  |
| CD8 n, % |  |  | 0.509 |  |  |  |  |
| Positive | 39 (65.0) | 16 (72.7) |  |  |  |  |  |
| Negative | 21 (35.0) | 6 (27.3) |  |  |  |  |  |
|  |  |  |  |  |  |  |  |
| Parameter | p16 positive | p16 negative | *p*-value |  |  |  |  |
|  | n = 60 | n = 22 |  |  |  |  |  |
| PD-1 n, % |  |  | 0.228 |  |  |  |  |
| Positive | 19 (31.7) | 4 (18.2) |  |  |  |  |  |
| Negative | 41 (68.3) | 18 (81.8) |  |  |  |  |  |
|  |  |  |  |  |  |  |  |
| Parameter | p16 positive | p16 negative | *p*-value |  |  |  |  |
|  | n = 60 | n = 22 |  |  |  |  |  |
| PD-L1 n, % |  |  | 0.854 |  |  |  |  |
| Positive | 12 (20.0) | 4 (18.2) |  |  |  |  |  |
| Negative | 48 (80.0) | 18 (81.8) |  |  |  |  |  |

Supplementary Table S3. Univariate and multivariate analyses of progression-free survival using a Cox proportional hazards model in patients with early-stage cervical adenocarcinoma

| Factor | Patients | Univariate analysis | | | Multivariate analysis | | |
| --- | --- | --- | --- | --- | --- | --- | --- |
|  | n = 72 | HR | 95% CI | *p*-value | HR | 95% CI | *p*-value |
| Age (years) |  |  |  |  |  |  |  |
| <60 | 57 | ref. |  |  | ref. |  |  |
| ≥60 | 15 | 1.089 | 0.307–3.860 | 0.895 | 7.742 | 1.296–46.255 | 0.025 |
| Histology |  |  |  |  |  |  |  |
| Non-gastric type | 59 | 0.572 | 0.182–1.799 | 0.34 | 0.592 | 0.114–3.068 | 0.532 |
| Gastric type | 13 | ref. |  |  | ref. |  |  |
| Tumor size (mm) |  |  |  |  |  |  |  |
| <40 | 47 | 0.097 | 0.027–0.344 | <0.001 | 0.01 | 0.001–0.146 | 0.001 |
| ≥40 | 25 | ref. |  |  | ref. |  |  |
| Metastasis Pelvic Lymph node |  |  |  |  |  |  |  |
| Negative | 57 | 0.282 | 0.102–0.780 | 0.015 | 0.192 | 0.045–0.832 | 0.027 |
| Positive | 15 | ref. |  |  | ref. |  |  |
| Metastasis Paraaortic Lymph node |  |  |  |  |  |  |  |
| Negative | 70 | 0.127 | 0.028–0.578 | 0.008 | ref. |  |  |
| Positive | 2 | ref. |  |  | 6.567 | 0.335–128.845 | 0.215 |
| Metastasis LSVI |  |  |  |  |  |  |  |
| No | 29 | 0.065 | 0.008–0.516 | 0.01 | 0.012 | 0.001–0.220 | 0.003 |
| Yes | 30 | ref. |  |  | ref. |  |  |
| p16^INK4A^ expression |  |  |  |  |  |  |  |
| Negative | 17 | ref. |  |  | 2.923 | 0.477–17.926 | 0.246 |
| Positive | 55 | 0.402 | 0.142–1.134 | 0.085 | ref. |  |  |
